# Tolerance for High Flavanol Cocoa Powder in Semisweet Chocolate

**DOI:** 10.3390/nu5062258

**Published:** 2013-06-21

**Authors:** Meriel L. Harwood, Gregory R. Ziegler, John E. Hayes

**Affiliations:** 1 Sensory Evaluation Center, Department of Food Science, College of Agricultural Sciences, The Pennsylvania State University, University Park, PA 16802, USA; E-Mail: mlh413@psu.edu; 2 Department of Food Science, College of Agricultural Sciences, The Pennsylvania State University, University Park, PA 16802, USA; E-Mail: grz1@psu.edu

**Keywords:** cocoa, cocoa flavanols, rejection thresholds, sensory evaluation, semisweet chocolate

## Abstract

Endogenous polyphenolic compounds in cacao impart both bitter and astringent characteristics to chocolate confections. While an increase in these compounds may be desirable from a health perspective, they are generally incongruent with consumer expectations. Traditionally, chocolate products undergo several processing steps (e.g., fermentation and roasting) that decrease polyphenol content, and thus bitterness. The objective of this study was to estimate group rejection thresholds for increased content of cocoa powder produced from under-fermented cocoa beans in a semisweet chocolate-type confection. The group rejection threshold was equivalent to 80.7% of the non-fat cocoa solids coming from the under-fermented cocoa powder. Contrary to expectations, there were no differences in rejection thresholds when participants were grouped based on their self-reported preference for milk or dark chocolate, indicating that these groups react similarly to an increase in high cocoa flavanol containing cocoa powder.

## 1. Introduction

Chocolate, a product of *Theobroma cacao*, is naturally bitter [1]. Chocolate contains phenolic compounds such as catechin, epicatechin, anthocyanins, and other various polyphenols [2]. Polyphenolic compounds are often perceived as both bitter and astringent [3], and an increase in polyphenol content has the potential to elicit stronger sensations of bitterness and astringency. For example, milk chocolate confections, which tend to have lower polyphenolic content [4] (and often higher sugar content) are generally less bitter and astringent than their semisweet (“dark”) counterpart, which is sometimes even referred to as “bitter chocolate”.

The process of fermentation reduces total polyphenol content in cacao in a time-dependent manner as these compounds can be oxidized, polymerized, or form complexes with other chemicals [5,6]. A previous study investigating the relative content of total polyphenols, tannins, and (−)-epicatechin has correlated these chemical contents with sensory panel-determined acceptability, indicating that “deficiently” fermented samples can have unacceptably high levels of tannins and (−)-epicatechin [5]. As such it is common practice to ferment cocoa beans to an optimal level for consumer acceptance, in addition to primary goals of fermentation such as seed death and formation of chocolate flavor precursors. However, there is interest in preserving the polyphenol content in foods [7], as these compounds are now being investigated for potential health benefits they may confer to the consumer [8]. Since bitterness and astringency are generally aversive to most consumers, the challenge now is to balance these oral sensations with phytonutrient content to create acceptable products [2].

One method for determining acceptable concentrations of compounds or ingredients that become aversive at high levels is to determine a group rejection threshold, e.g., [9,10,11,12,13,14,15]. Use of rejection thresholds were originally applied to “off flavors” in wines, e.g., [9,11,15]. Subsequently, this method has also been used with other liquid and solid food products, e.g., [10,12,13]. The rejection threshold method, which is generally carried out as an ascending series of paired preference tests, allows investigators to determine the concentration at which an ingredient or taint leads to rejection of a spiked sample relatively to a control sample. This method is ideal for investigating the consumer response to chocolate formulated with greater levels of putatively heart healthy compounds, which generally elicit aversive astringent and bitter sensations, relative to chocolate formulated with traditionally processed cocoa. The rejection threshold method allows investigators to work with naïve consumers as opposed to trained panelists, which enables research into consumers’ initial, uninformed responses to products, similarly to how they may react when trying a new product for the first time.

Previous studies investigating rejection thresholds for bitterness in chocolate flavored products [12,13,14] have relied on an added bitter ingredient (sucrose octaacetate, or SOA) that is not a naturally occurring source of bitterness in chocolate. While these studies provide important theoretical groundwork, it remains to be seen if the same clear segmentation will occur in similar populations when the differences between the samples are due to bitter and astringent compounds normally found in chocolate. That is, previous studies have found that populations preferring dark chocolate have significantly higher rejection thresholds for the added bitterant (SOA) when presented in chocolate-flavored products such as chocolate milk [12], milk chocolate-flavored compound coating [13], and chocolate ice cream [14] when compared to populations that prefer milk chocolate. The primary aim of this study was to determine group rejection thresholds for increased content of cocoa powder produced from under-fermented cocoa beans (and therefore increased cocoa flavanol content) in a semisweet chocolate-type confection, and to compare rejection thresholds when participants were grouped based on their self-reported preference for milk chocolate or dark chocolate.

## 2. Materials and Methods

### 2.1. Test Stimuli

The test stimuli were semisweet chocolate pieces produced from the ingredients described below. High Cocoa Flavanol Natural Cocoa Powder, 10%–12% fat content, produced from under-fermented cocoa beans, was donated by Mars, Inc. (Elizabethtown, PA, USA). The high flavanol content of this cocoa powder, a result of the reduced fermentation, makes this cocoa powder more bitter and astringent than traditionally processed cocoa powder, and when tasted in isolation gives an overall complex impression of bitterness, astringency, and chocolate character. This cocoa powder will be referred to as “high CF cocoa powder” from here on. NI Natural Cocoa Powder (10%–12% fat content), which is less bitter than the under-fermented cocoa powder, was chosen as the “control” cocoa powder, and donated by Blommer Chocolate Company (Chicago, IL, USA). This cocoa powder will be referred to as “NI natural cocoa powder” from here on. Cocoa butter and soy lecithin were donated by the Barry Callebaut Chocolate Company (Pennsauken, NJ, USA). Additional ingredients include sucrose (Good Food Inc., Honey Brook, PA, USA) and canola oil (Wegmans Food Markets, Inc., Rochester, NY, USA). We will refer to the samples as semisweet chocolate for the remainder of this manuscript, as they exceed the minimum liquor content requirement for semisweet chocolate in the United States. However, we should note that the use of reconstituted cocoa liquor rather than liquor ground directly from cocoa beans, and the addition of a very small amount of canola oil, means that the samples do not meet the legal standard of identity (21 CFR 163.123) [16] for sale as semisweet chocolate in the United States. Canola oil (an allergen-free fat liquid at room temperature) was added to slightly soften the samples to make them easier to eat (similar to the role of dairy fat [17]).

Semisweet chocolate samples were prepared containing different proportions of the cocoa powders. These samples were prepared from a refined cocoa butter and sugar flake base; reconstituted liquors made from the NI natural and high CF natural cocoa powders with added cocoa butter; canola oil and soy lecithin. The proportion of components in the samples is outlined in Table 1. All of the non-fat cocoa solids were added at the conch, with additional cocoa butter, canola oil, and soy lecithin. The different samples contained increasing proportions of high CF natural cocoa powder relative to NI natural cocoa powder (outlined in Table 2). The cocoa butter/sugar base was mixed, refined, and then divided into the different batches. The batches were conched (uncovered at 65 °C for 4 h), tempered, molded, and stored in tightly sealed containers away from light at room temperature (22 °C) for ten days before sensory testing. Each piece weighed approximately 2.5 g, and each participant received one piece of chocolate of each of the five concentrations, paired with a piece of the control chocolate (containing only NI natural cocoa powder), resulting in a total of ten pieces per participant. While these samples were quite small, rejection thresholds have previously been successfully determined in smaller (0.63 g) solid milk chocolate-flavored compound coating samples spiked with the bitter compound sucrose octaacetate [13]. Samples were presented in clear plastic cups with lids labeled with random, three-digit blinding codes.

**Table 1:** Sample formulation. Percentage of each component comprising the sample formula for the semisweet chocolate used.

Components	Percentage
Non-fat cocoa solids	44%
Fat *	32%
Sugar	23.5%
Lecithin	0.5%

* 29% cocoa butter, 3% canola oil.

**Table 2:** Proportions of cocoa powders in test stimuli. Proportions of NI natural cocoa powder and high CF natural cocoa powder in each of the spiked samples as well as the control samples.

Log concentration	NI natural cocoa powder	High CF natural cocoa powder
*Control*	100%	0%
*1.55*	65%	35%
*1.70*	50%	50%
*1.80*	35%	65%
*1.90*	20%	80%
*2.00*	0%	100%

### 2.2. Sensory Testing Procedures

Ninety-nine chocolate consumers (thirty-seven men) were recruited from the Penn State community via email based on their liking of and willingness to consume chocolate. All participants were reportedly healthy, non-smoking individuals aged between 18 and 45 years. Fifty-three of the participants reported preferring milk chocolate and forty-six reported preferring dark chocolate. Under the wholesome foods/approved food additives exemption in 45 CFR 46.101(b)(6) all procedures were exempt from Institutional Review Board review by the Penn State Office of Research Protections. Participants in this study provided informed consent and were compensated for their time.

All tests occurred in the individual testing booth in the Sensory Evaluation Center at Penn State under white lighting, and Compusense *five* v5.2 (Guelph, ONT, Canada) was utilized for data collection. All data were collected in one testing session with 2-Alternative Forced Choice (2-AFC) preference tasks to determine rejection thresholds preceding demographic questions (age, gender, milk or dark chocolate preference). Semisweet chocolate samples were presented as pairs containing one control sample (100% NI natural cocoa powder) and one “spiked” sample containing high CF natural cocoa powder for a total of five pairs. The spiked samples were presented in order of increasing content of high CF natural cocoa powder and the presentation order within the pairs was randomized. Participants were instructed to eat each entire sample, and rinse with room temperature (22 °C) water between samples.

### 2.3. Total Phenolic Content

The phenolic extraction procedure used here was adapted from the protocol used by Hammerstone and colleagues [18]. Twenty (20) g of each cocoa powder were defatted with hexane three times and left to air dry. Yields of defatted cocoa powder were weighed and calculated as a fraction of the original wet weight. Polyphenols were then extracted from the samples with acetone and water (70:30) twice and methanol and water (50:50) twice. Organic solvents were removed by rotary evaporator under partial vacuum at ~28 °C. Samples were then freeze-dried. Polyphenol content of the freeze-dried extracts was then quantified by the Folin-Ciocalteu method [19].

### 2.4. Statistical Analysis

Rejection thresholds were analyzed with a sigmoid fit using the Hill Equation as previously described [12]. Group rejection thresholds were calculated for the entire sample population as well as for the group of individuals within this population who reported preferring milk chocolate, the group of individuals who reported preferring dark chocolate, and on the basis of gender. Associations between gender and solid chocolate preference were analyzed using Fisher’s Exact Test (two tailed).

## 3. Results and Discussion

**Figure 1:**
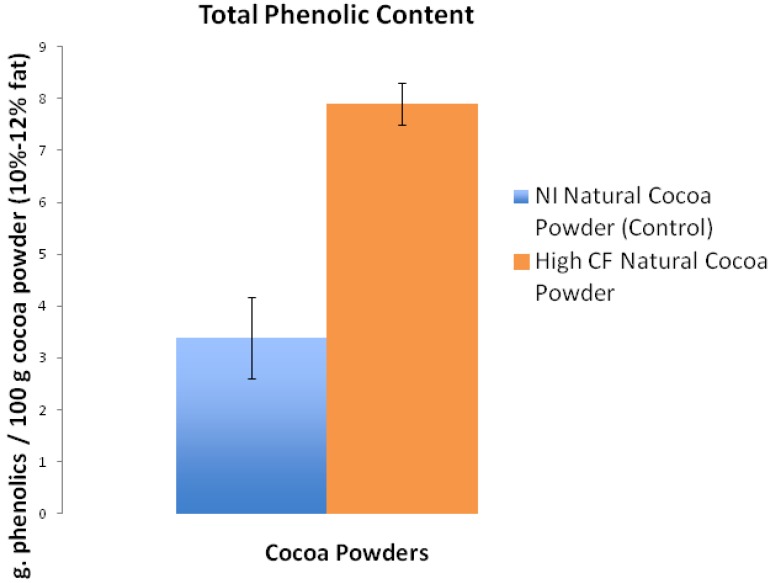
Relative phenolic content in 10%–12% fat cocoa powder. Grams of phenolic compounds per 100 g of cocoa powder (NI natural and high CF natural) determined by Folin-Ciocalteu with standard deviation.

Defatting the cocoa powder samples with hexane yielded 15.85 g fat free cocoa solids from the NI natural cocoa powder and 16.62 g fat free cocoa solids from the high CF natural cocoa powder. The final yield was 2.83 g of freeze-dried extract from the NI natural cocoa powder and 4.50 g of freeze-dried extract from the high CF natural cocoa powder. The total phenolic assay revealed the phenolic content of the NI natural cocoa powder to be 3.4% w/w (g phenolic per 100 g of cocoa powder, 10%–12% fat) compared to 7.9% w/w for the high CF natural cocoa powder; a 2.3-fold difference (Figure 1). As bitter and/or astringent taste components have been shown to increase with polyphenol content in other studies, e.g., [20], this result (the higher phenolic content of the high CF natural cocoa powder) confirms the decision to use these different cocoa powders to formulate the semisweet chocolate for the sensory portion of this experiment. However, descriptive profiling would still be required to make any specific conclusions on which sensory attribute from the increasing level high CF natural cocoa powder led to eventual rejection of the test stimuli. While we did not measure the total phenolic content of these samples after processing (conching and tempering especially as these involve heat) the samples were all subjected to the same processing conditions, so we would expect them to contain the same relative proportions of polyphenols, as any loss would be equivalent across all of the samples.

A significant association was found between gender and solid chocolate preference in this study (*p* = 0.0126). Of the fifty-three participants who reported preferring milk chocolate, twenty-six were male and twenty-seven were female. In contrast, of the forty-six participants who reported preferring dark chocolate, only eleven were male and thirty-five were female. This could potentially represent a limitation in this study, as dark chocolate preferring men were under-represented. However, there were no significant differences found in the rejection thresholds when comparing the men and the women in this study (*p* = 0.803).

**Figure 2:**
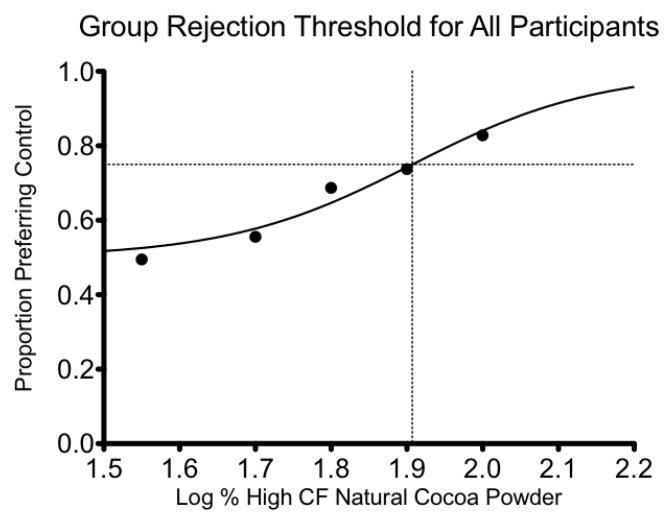
Preference/indifference Function for all participants combined. The preference/indifference function across all participants (*n* = 99) for high CF natural cocoa powder in semisweet chocolate. *Y*-axis shows the proportion of respondents choosing the control sample, and the *x*-axis shows the concentration of high CF natural cocoa powder in the spiked samples. The group rejection threshold is defined at the concentration at which the fitted curve crosses 0.75 on the *y*-axis. This criterion (75%) represents the proportion halfway between chance performance (50%) and universal rejection (100%). See [9] for more information.

The group rejection threshold for the high CF natural cocoa powder in semisweet chocolate for all of the participants together was 80.7% (see Figure 2), falling almost exactly at the second highest spiked sample. Further analysis revealed no significant differences (*p* = 0.6235) between the rejection threshold for the group preferring milk chocolate and the group preferring dark chocolate. This suggests that regardless of reported preference for milk or dark chocolate, all of the participants reacted in a similar manner to the increased content of under-fermented cocoa powder in semisweet chocolate. An interesting characteristic of the preference/indifference function in Figure 2 is the shallow slope of the linear portion. The increase in rejection across concentrations is very gradual when compared to other measured rejection thresholds [12,13] obtained with the bitter compound SOA.

One potential reason for the lack of segmentation observed between milk chocolate and dark chocolate preferring groups could be that the predominant difference in the samples was not the same characteristic that milk or dark chocolate preference is based on. That is, it seems reasonable that the bitterness and astringency of dark chocolate may be what milk chocolate preferring individuals find aversive about dark chocolate. However, it is unknown whether bitterness or astringency was the predominant character setting the spiked samples apart from the controls in the present experiment, as we did not directly assess these attributes. Nonetheless, even without quantifying bitterness and astringency directly, it seems likely that increased polyphenol content from high CF cocoa power eventually results in rejection. This would seem to be a strength of the method, in that the objectionable attribute need not be known to objectively determine how much of an ingredient is too much. That said, it also remains possible that the spiked samples simply had less desirable “chocolate flavor” due to less fermentation and hence were less preferred, as the forced preference task only implies that one is more preferable compared to the other. It is important to note that with rejection thresholds, the driver of rejection could potentially be the absence of a positive attribute. The 2-AFC task forces participants to select one sample over another within a pair, meaning preferences are always relative to the other sample. Rejection thresholds are a method of constant stimuli; across pairs, increasing concentrations of the spiked sample are compared to a control sample that is constant. There are many qualities that differentiate milk and dark chocolate, varying from flavor (e.g., sweetness and dairy notes) to melting quality. Training a panel to create a descriptive profile of the spiked and control samples and exploring this further may have shed more light on the reasons for the lack of differences in the rejection of the spiked samples. Still, rejection thresholds appear to provide actionable information regarding formulation even in the absence of information from descriptive profiling.

Notably, it remains possible that the failure to observe segmentation here was due to the criteria on which the participants were grouped. Participants were asked, “When you consume solid chocolate, which do you prefer: milk chocolate or dark chocolate?” and categorized on this basis. There are a wide variety of products, with highly variable sensory qualities that can be categorized as milk chocolates or dark chocolates. As such, we may have missed an effect that may have been seen more clearly if we had gathered additional information about these participants’ chocolate preferences, such as cacao content or brand. Also it is possible that some participants enjoy both milk chocolate and dark chocolate, without showing strong preference of one over the other, but were forced to choose due to the way the question was asked, blunting differences that may have been otherwise observed between staunch milk chocolate or dark chocolate likers.

Additionally, while we can infer tolerance from the rejection threshold measure, it remains to be seen how the participants would rate their liking of the samples presented here. The base formula for the samples is similar to other commercially available products. For example, Lindt Excellence 70% Cacao chocolate, which contains cocoa mass, sugar, cocoa butter, and natural bourbon vanilla beans, contains a similar amount of cacao (70% compared to the 73% cacao content of the study samples), though slightly more fat (40% fat in Lindt compared to 32% fat in the study samples) and sugar (26.6% sugar in Lindt compared to 23.5% sugar in the study sample) [21]. Therefore it may be reasonable to assume that the control sample, which contained traditionally processed cocoa powder, would be rated at least as acceptable. Notably, a sample that is “rejected” may not actually be objectionable, as rejection is inferred from a forced choice task. It may be that the rejected sample is highly liked, just less so than the other sample in the pair.

## 4. Conclusions

The rejection threshold for high CF natural cocoa powder in this semisweet chocolate-type confection is 80.7%. No significant difference in rejection threshold was observed when grouping participants based on their self-identified preference for milk or dark chocolate. While solid chocolate preference (milk *vs*. dark) has been successfully applied to differences in rejection thresholds for a bitter compound previously [12,13], this segmentation strategy does not appear to be appropriate when considering tolerance for increased content of high CF natural cocoa powder in semisweet chocolate-type confections, perhaps due to the complexity inherent to chocolate flavor.
